# Visual Strategies for Eye and Head Movements During Table Tennis Rallies

**DOI:** 10.3389/fspor.2022.897373

**Published:** 2022-05-17

**Authors:** Ryosuke Shinkai, Shintaro Ando, Yuki Nonaka, Tomohiro Kizuka, Seiji Ono

**Affiliations:** ^1^Graduate School of Comprehensive Human Sciences, University of Tsukuba, Ibaraki, Japan; ^2^Faculty of Health and Sport Sciences, University of Tsukuba, Ibaraki, Japan

**Keywords:** eye movements, head movements, gaze, table tennis, visual strategy

## Abstract

The purpose of this study was to clarify the properties of visual strategies for gaze, eye, and head movements in skilled table tennis players during rallies. Collegiate expert and semi-expert table tennis players conducted forehand rallies at a constant tempo using a metronome. Two tempo conditions were used in the order of 130 and 150 bpm. Participants conducted a 20-stroke rally under each tempo condition. Horizontal and vertical angles between the gaze point and ball positions at the time the ball bounced (gaze-ball angle) were analyzed with the image that was recorded by an eye tracking device equipped with Gyro sensor. Eye and head movements during rallies were also recorded with the eye tracking device and Gyro sensor, respectively. The results showed that the gaze-ball angle of expert players was significantly larger than that of semi-expert players. This result indicates that expert players tended to keep their gaze position on the ball shorter than semi-expert players. We also found that eye movements of expert players were significantly smaller than that of semi-expert players. Furthermore, as the result of multiple regression analysis, the effect of eye movements on the gaze-ball angle was significantly higher than that of head movements. This result indicates that the gaze-ball angle during table tennis rallies could be associated with eye movements rather than head movements. Our findings suggest that the visual strategies used during table tennis rallies are different between expert and semi-expert players, even though they both have more than 10 years of experience.

## Introduction

Table tennis is a sport in which players face each other across a 2.74-meter-long court and hit a ball at each other. One of the characteristics of table tennis is that the hitting ball speed feels relatively fast as in other sports despite the relatively small court size. In fact, it has been reported that table tennis players have a better ability to adjust their timing to fast-moving visual targets than badminton or tennis players (Akpinar et al., 2012). Eye-hand coordination is also recognized as one of the important factors in table tennis (Faber et al., 2014). The studies mentioned above suggest that a higher level of visual cognitive ability is required through daily skill training in table tennis. In addition, the speed of the racket hitting a ball is thought to be one of the important factors to improve competitive performance (Kidokoro et al., 2019). It has also been reported that the higher the competition level, the higher the hitting ball speed (Sheppard and Li, 2007; Mansec et al., 2016). Thus, it is considered that the higher the competition level of the opposite player, the more instantaneous visual perception is required during rallies. Therefore, skilled table tennis players would have a specific visual strategy to ensure successful performance under severe time constraints during fast rallies. In the present study, visual strategy is defined as how to control gaze position with eye and head movements.

Ripoll and Fleurance (1988) have indicated that it is not necessary to keep one's eyes on the ball throughout the entire trajectory during the ball-tracking in table tennis because of the severe time constraints during table tennis rallies. Moreover, Ishigaki (2007) has reported that the visual strategy during table tennis rallies at a constant tempo differs between competition levels. Concretely, the higher the competition level, the earlier the player shifts his eyes from the ball to the opposite player. These findings are consistent with those of Ripoll and Fleurance (1988). However, in the previous study of Ishigaki, a high-speed camera was used to estimate the gaze point rather than an eye-tracking device. Moreover, there were few participants as the maximum number of participants was one or two in each skill group. Thus, it is necessary to analyze the detailed gaze patterns during table tennis rallies between different competition levels to clarify the property of visual strategy.

Regarding cricket batting, Mann et al. (2013) have used the gaze-ball angle as one of the parameters to examine the difference in visual strategy between world level and club level players. The gaze-ball angle is defined as the angle between the gaze point and the ball during the ball tracking phase. Their study has found different visual strategies when the ball bounces between two subject groups. Although the angle between the gaze point and the ball at the time the ball bounces is one of the phases in ball tracking, this angle may reflect the characteristics of gaze position during the ball tracking. Rodrigues et al. (2002) have also calculated the gaze-ball angle during the ball-tracking in table tennis. However, this angle was only used to define the “Quiet eye” and was not assessed between different skill levels. Therefore, we attempted to assess the gaze-ball angle at the time the ball bounces to clarify the visual strategy during table tennis rallies between different competition levels.

Furthermore, it is important to evaluate not only gaze behavior but also eye and head movements since gaze behavior is determined by eye-head interactions (Roy and Cullen, 2003; Pallus and Freedman, 2016). Therefore, if gaze behavior is different between competition levels, the eye and head movements related to gaze behavior may also show different properties. That may reveal differences in detailed visual strategies that cannot be assessed only by the gaze behavior alone. Regarding eye and head movements during intercept performance, it has been reported that skilled players do not move their head before or after hitting (LaFont, 2007; Nakata et al., 2012) and that stable and minimal eye movements are important in ball tracking for skilled baseball batters (Higuchi et al., 2018). These findings suggest that the properties of eye and head movements during the interceptive performance are different depending on the competition level. The control of eye and head movements would be an effective gaze strategy during intercept performance. However, it is still uncertain whether eye and head movements contribute differently to gaze behavior during table tennis rallies.

Therefore, the purpose of this study was to clarify the properties of the visual strategies for the gaze-ball angle, eye and head movements in skilled table tennis players during table tennis rallies. In this study, we quantified not only the horizontal components of gaze-ball angle, eye and head movements but also their vertical components. The mean angular velocity was utilized to quantify the participant's visual strategy to examine the properties of the eye and head movements. These data were compared with two groups, including expert and semi-expert table tennis players.

## Materials and Methods

### Participants

The participants were thirteen college students belonging to a table tennis team with a mean age of 21.4 years (range 18–23) and they reported having normal or corrected to normal vision and no known motor deficits. We categorized them into two groups (experts and semi-experts) based on their tournament experience. Six male players who participated in the All-Japan tournament (mean age: 20.8 ± 1.2 years, table tennis experience: 13.8 ± 1.7 years, height: 170.9 ± 6.8 cm, body mass: 63.3 ± 6.1 kg, five right-handed and one left-handed) were defined as an expert group, and another seven male players who had more than 10 years of experience but had no tournament experience (mean age: 21.9 ± 0.8 years, table tennis experience: 10.1 ± 1.1 years, height: 171.0 ± 3.3 cm, body mass: 66.2 ± 5.7 kg, six right-handed and one left-handed) were defined as the semi-expert group. They were diagnosed neither as a stereoscopic problem nor strabismus. All participants gave their informed consent to participate in the experiment.

### Experimental Procedure

**Figure 1:**
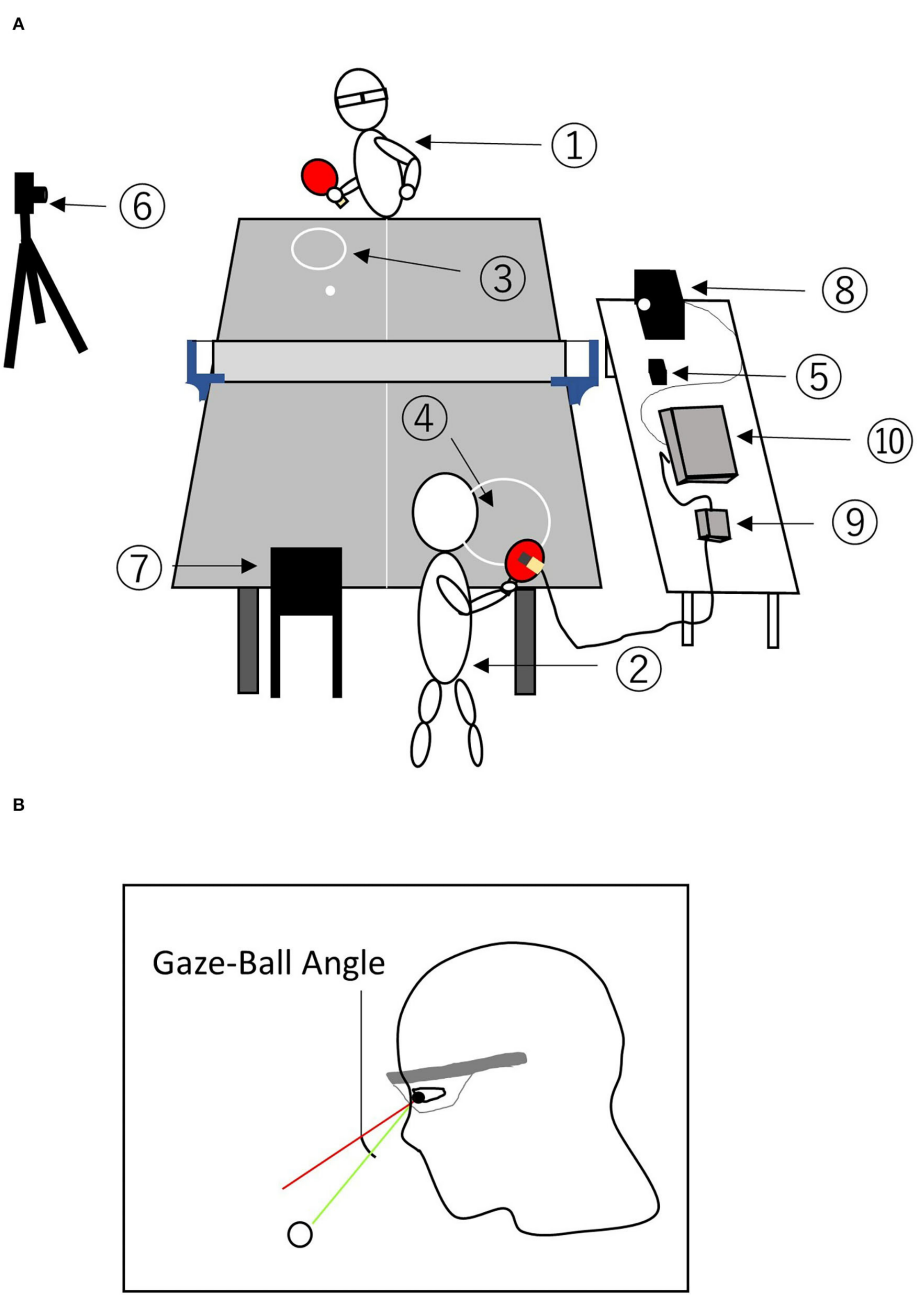
Overview of experimental task **(A)** and measurement of the gaze-ball angle **(B)**. **(A)** ① participant, ② skilled experimenter, ③ participant-side circle targets, ④ experimenter-side circle target, ⑤ speaker, ⑥ high speed camera, ⑦⑧ LED light, ⑨ control box for gyro sensor, ⑩ waveform generator. **(B)** The gaze position coordinates were subtracted from the ball position coordinates to estimate the gaze-ball angle.

**Figure 2:**
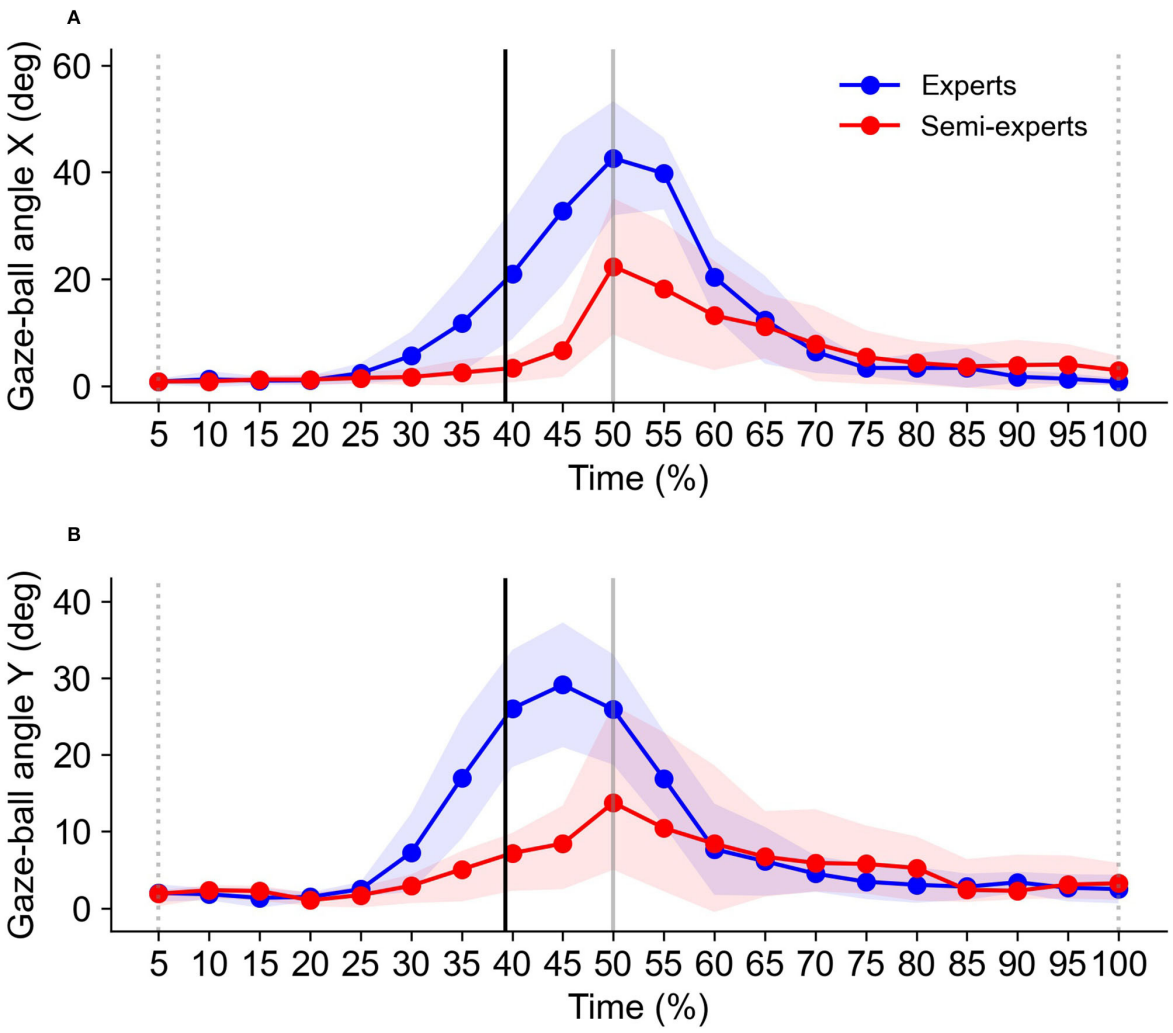
Typical waveform patterns of the gaze-ball angle in X **(A)** and Y **(B)** directions. The broken gray lines indicate the time when the experimenter hits the ball, and the solid gray line indicates the time when the participant hits the ball. The solid black line indicates the mean time when the ball bounced.

**Figure 3:**
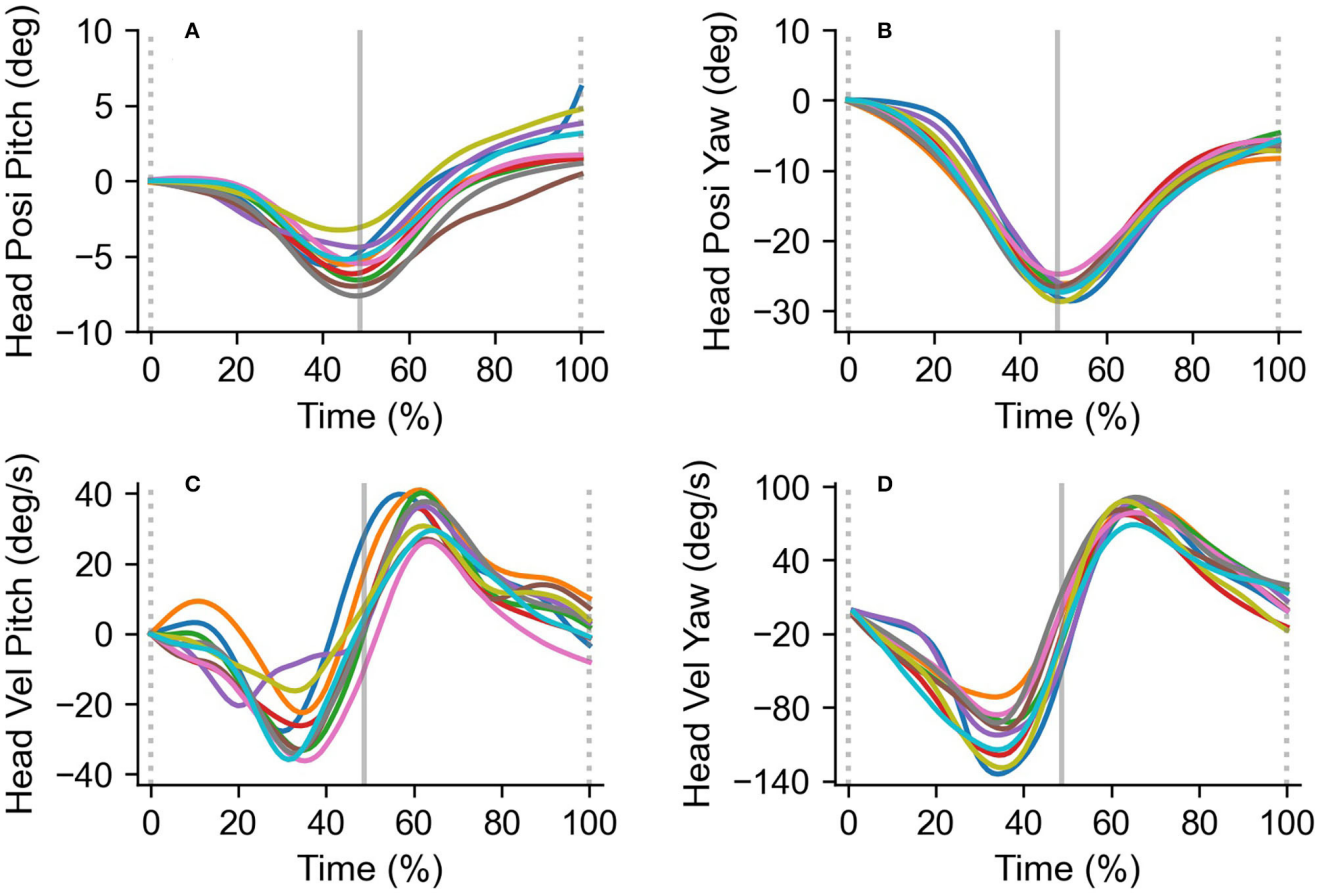
Typical head angular position and velocity traces in the pitch (left column) and yaw (right column). Head angular position in the pitch **(A)** and yaw **(B)**, and head angular velocity in the pitch **(C)** and yaw **(D)** axes are shown. All traces were obtained from a single trial (10 rallies a trial). The broken lines indicate the time when the experimenter hits the ball, and the vertical solid lines indicate the time when the participant hits the ball. The downward and upward velocity phases correspond to the ball tracking and hitting phases, respectively. Upward deflections indicate upward or leftward movements.

**Figure 4:**
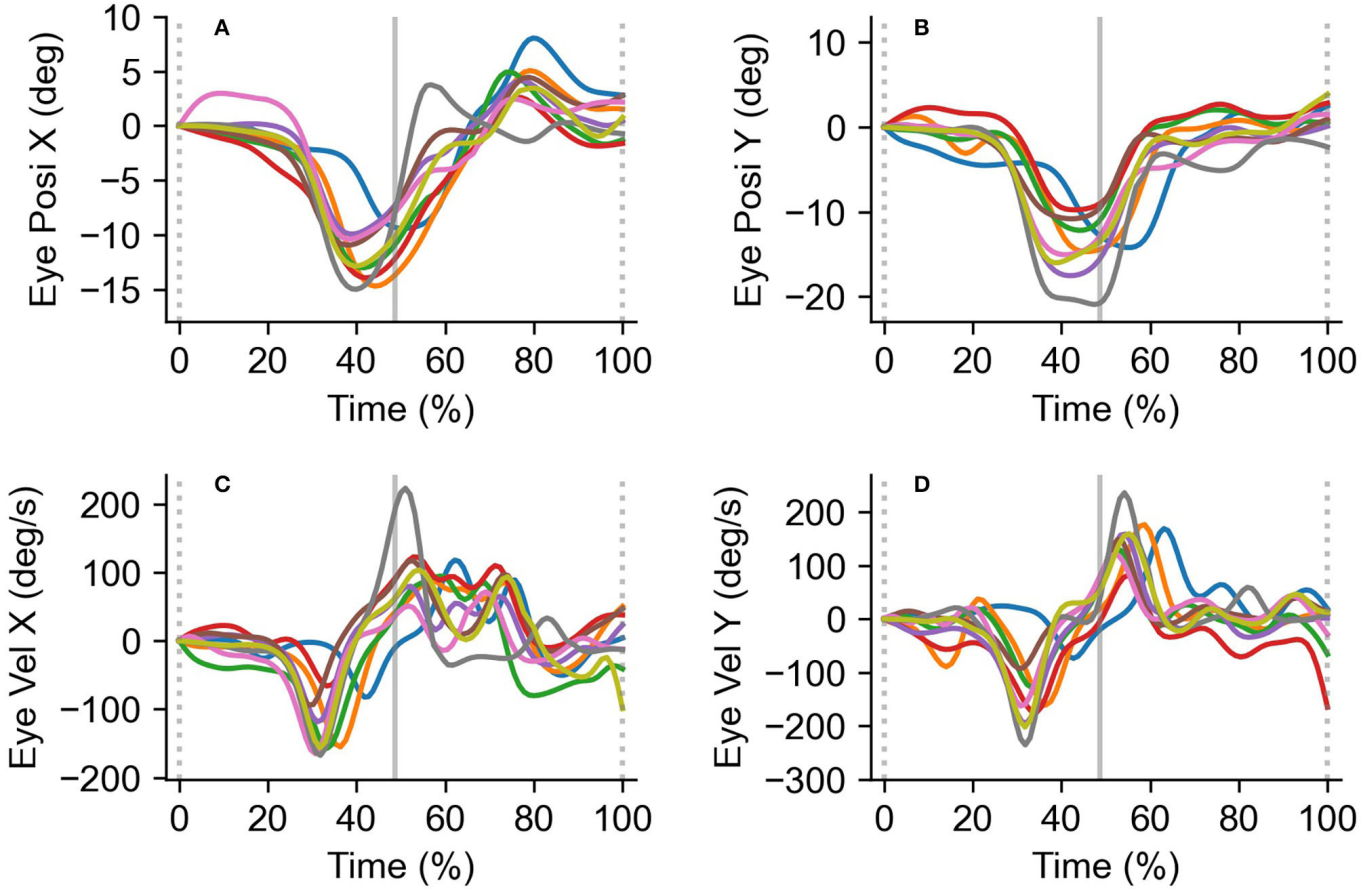
Typical eye position (top) and velocity (bottom) traces. Eye position in the horizontal **(A)** and vertical **(B)** directions, and eye velocity in the horizontal **(C)** and vertical **(D)** directions are shown. All traces were obtained from a single trial (10 rallies a trial). The definition of the vertical lines is the same as in Figure 3. Upward deflections indicate upward or leftward movements.

**Figure 5:**
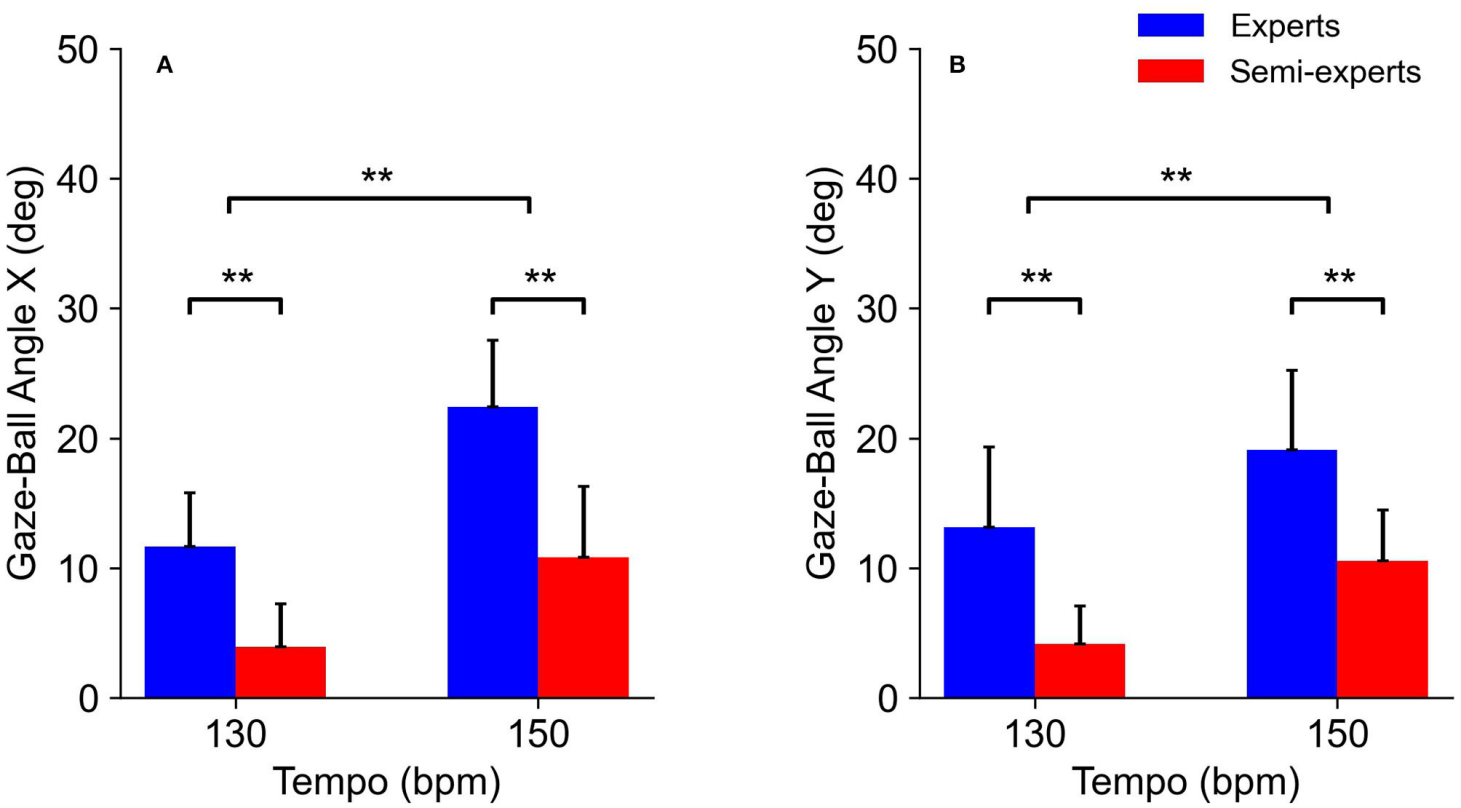
Comparison of the gaze-ball angle X **(A)** and Y **(B)** of the expert and semi-expert groups in the two tempo conditions.

Participants wore an eye-tracking device (Tobii Pro glasses 2, Tobii Technology, Stockholm) to record eye movements during experimental tasks, and the calibration was performed before the experiment. Head movement was recorded by the built-in gyro sensor (frequency: 100 Hz) with the eye-tracking device. The experimenter (Figure 1A-①) conducted the forehand rallies as experimental tasks with participants (Figure 1A-②). The experimenter delivered a ball to one target (diameter: 24 cm, Figure 1A-③) on the participant's side, and the participant would aim to hit the ball to the circular target (diameter: 30 cm, Figure 1A-④) on the experimenter's side. Participants conducted several trials to become familiar with this task. A metronome speaker (Creative MUVO 2c, CREATIVE, Japan, Figure 1A-⑤) was set on the table near the net to accurately control the timing of the hits. The two tempo sound conditions were conducted in the order of 130 and 150 bpm. In each tempo condition, they conducted a total of 20 shots (two trials). The previous study by Ishigaki (2007) set 120 bpm for regular tempo rallies. This tempo condition shows that one rally is conducted in just 1 s. In this study, we wanted to examine visual strategy at faster tempo thresholds. Therefore, we used 130 and 150 bpm during the rallies, since this tempo indicates one rally <1 s (130 bpm, 0.92 s/rally; 150 bpm, 0.8 s/rally). The experimenter was the same person in all experiments.

**Figure 6:**
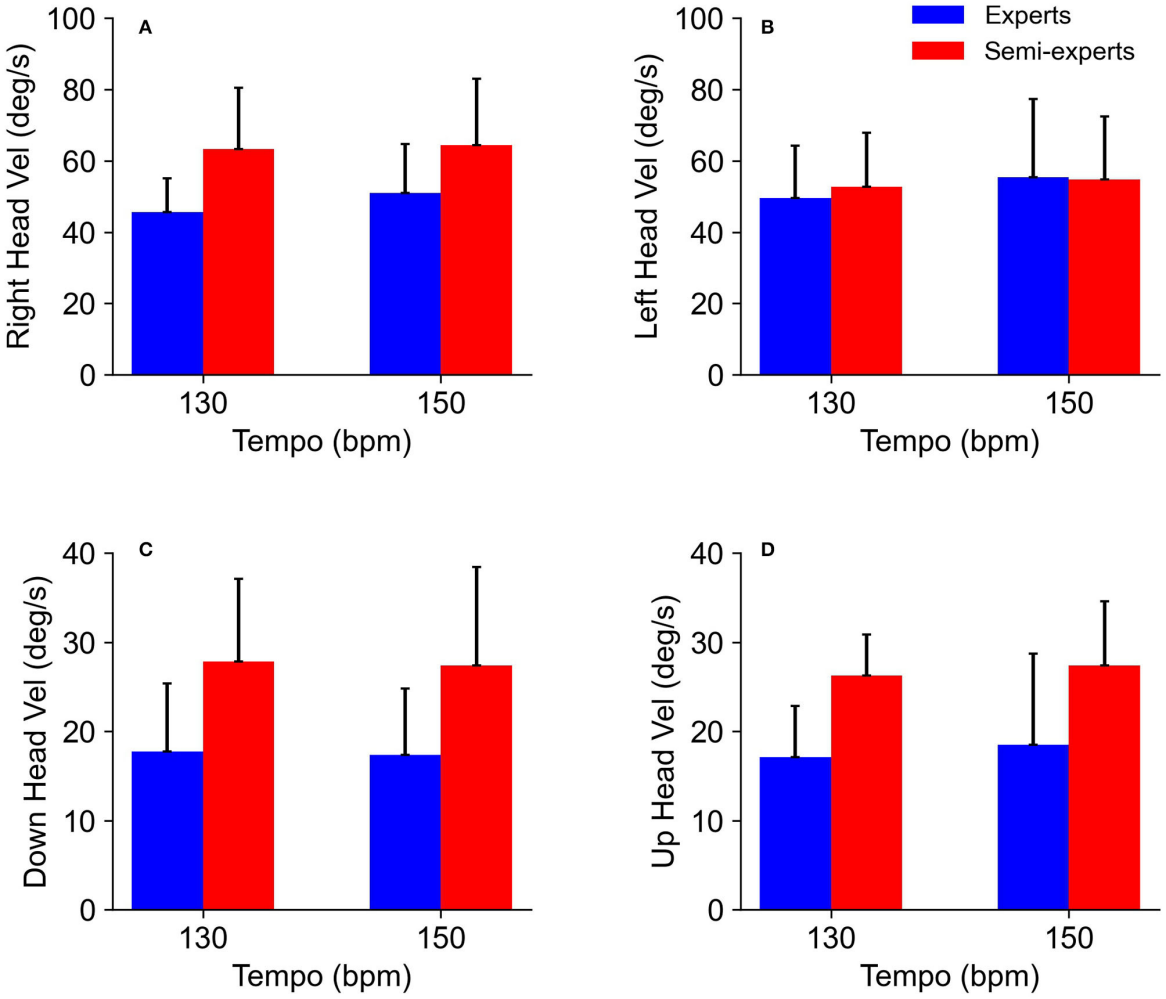
Comparison of the head velocity of the expert and semi-expert groups in the two tempo conditions. **(A)** Comparison of the rightward head velocity (right head vel), **(B)** leftward head velocity (left head vel), **(C)** downward head velocity (down head vel) and, **(D)** upward head velocity (up head vel) between the experts and semi-experts in the two tempo conditions are shown.

**Figure 7:**
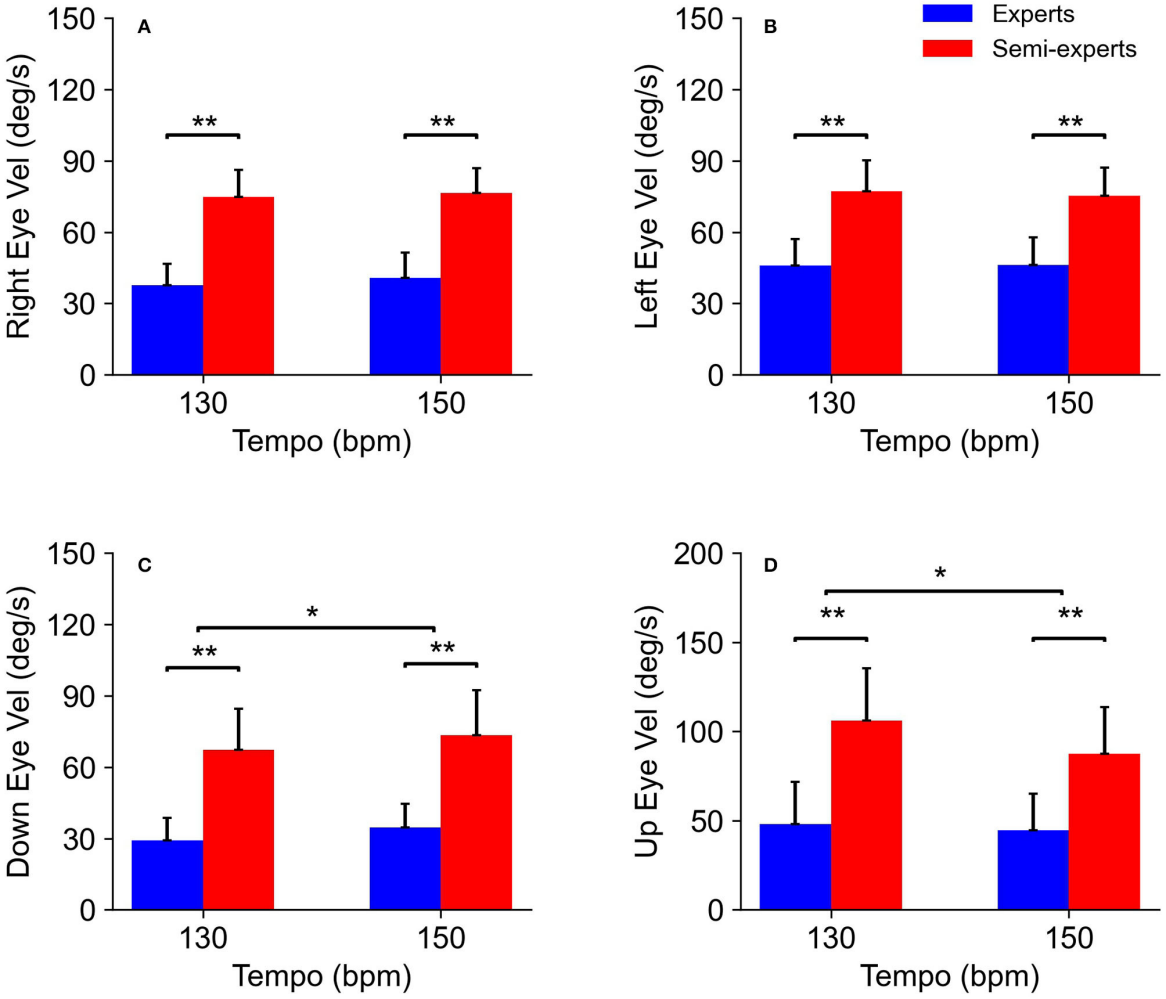
Comparison of the eye velocity of the expert and semi-expert groups in the two tempo conditions. **(A)** Comparison of the rightward eye velocity (right head vel), **(B)** leftward eye velocity (left head vel), **(C)** downward eye velocity (down head vel) and, **(D)** upward eye velocity (up head vel) between the experts and semi-experts in the two conditions are shown.

**Figure 8:**
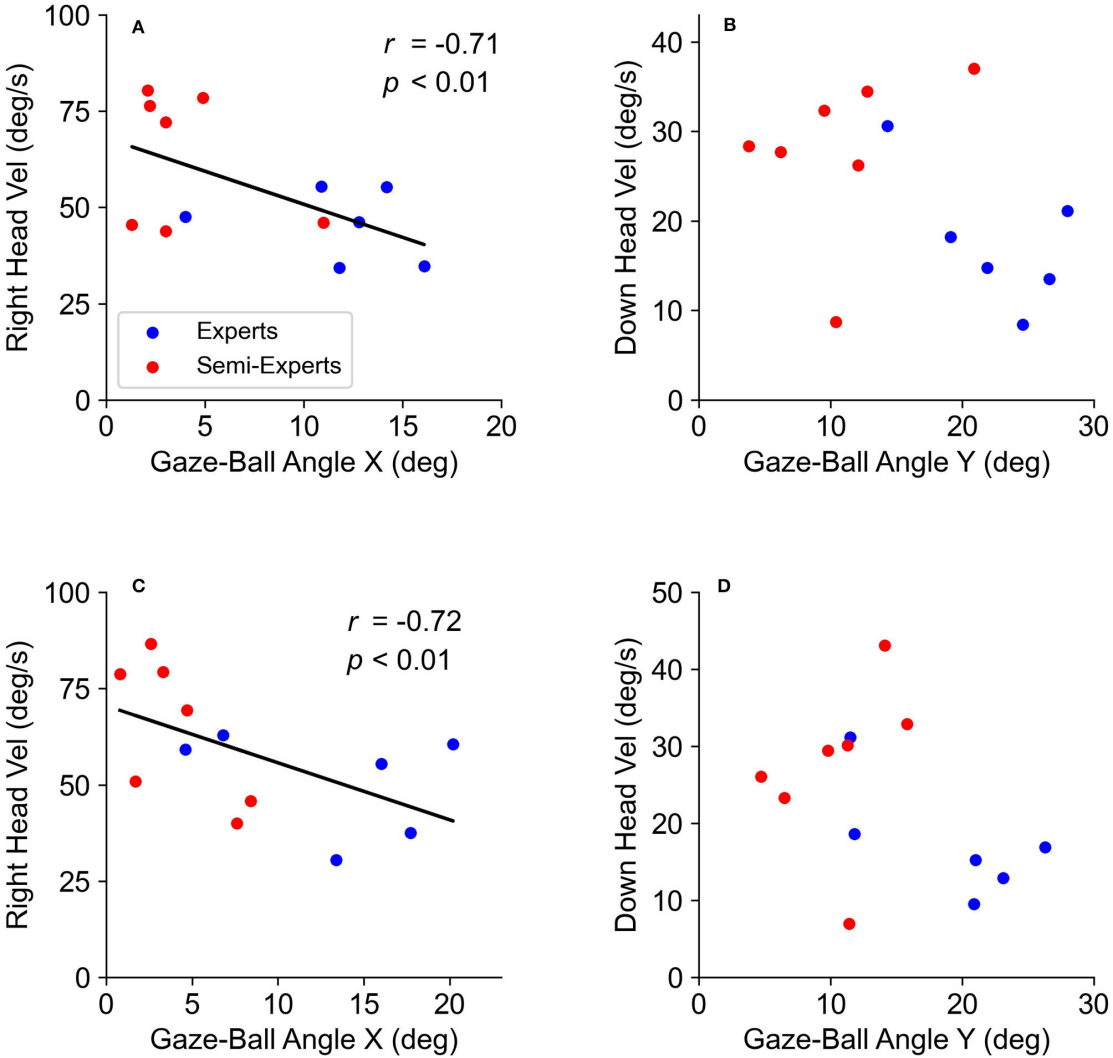
The relationship between the gaze-ball angle and head velocity in each tempo condition. The top two panels **(A,B)** show the data at 130 bpm. The bottom two panels **(C,D)** show the data at 150 bpm.

**Figure 9:**
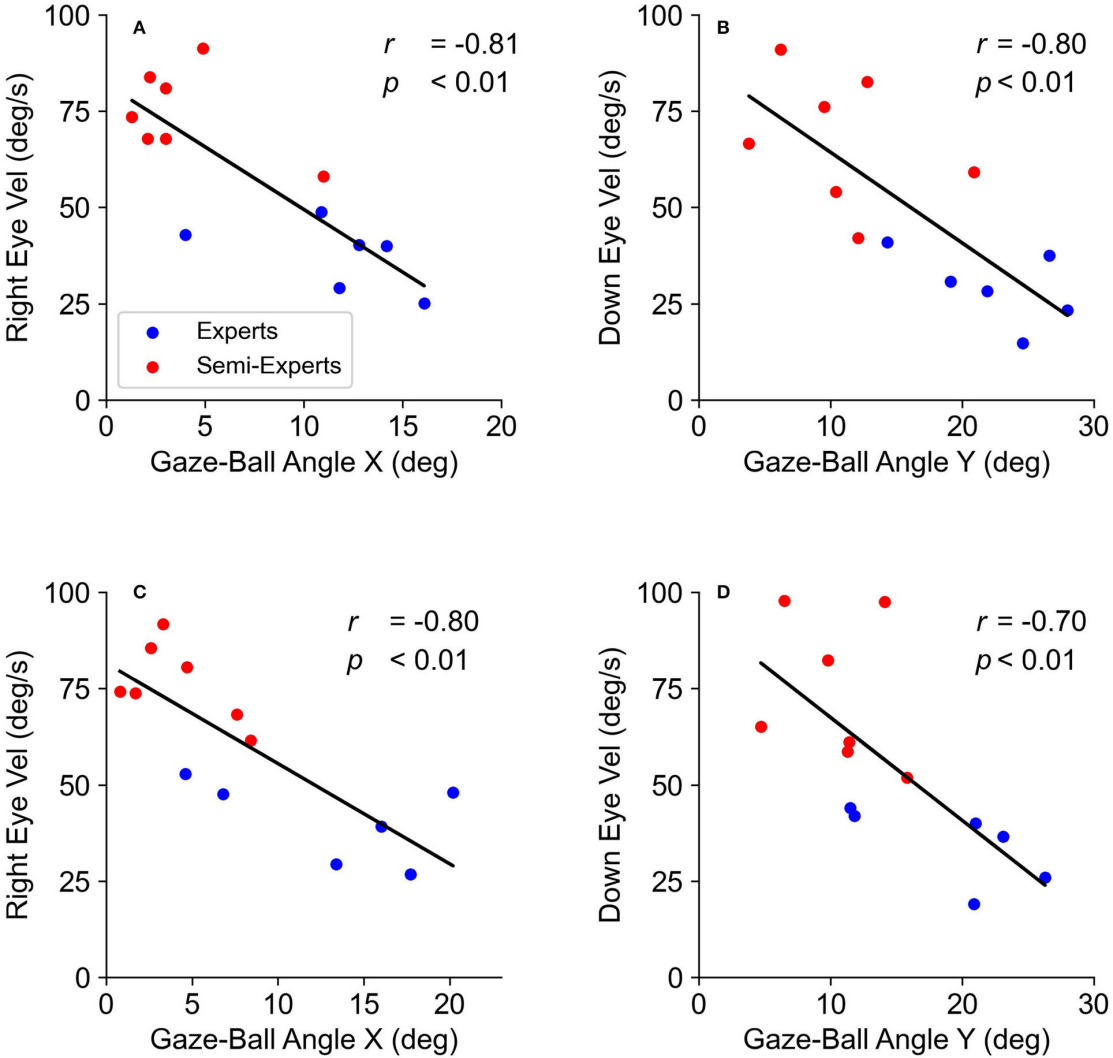
The relationship between the gaze-ball angle and eye velocity in each tempo. The top two panels **(A,B)** show the data at 130 bpm. The bottom two panels **(C,D)** show the data at 150 bpm.

To synchronize the impact time of the scene camera of the eye-tracking device and the high speed camera (Figure 1A-⑥), an acceleration sensor was attached to the rear of the racket of the experimenter, and the LED lights (Figure 1A-⑦,⑧,) were set to provide the signal output from the acceleration sensor (MP-A0-01A, MicroStone, Japan, Figure 1A-⑨) through the waveform generator (SG-4211, IWATU, Japan, Figure 1A-⑩). Therefore, the LED lights were flashed by the vibration at the moment of the experimenter's impact. The delay from the hitting time to LED flash was <10 ms. The time when the LED light (Figure 1A-⑧) flashed was captured from each image of the high-speed camera (frame rate = 120 fps, EX-ZR200, CASIO, Japan, Figure 1A-⑥). The high-speed camera was set to reflect the LED flash (Figure 1A-⑧) meant the impact time of the experimenter and the stroke movement of participants. Therefore, we could capture the impact time of both players accurately. Furthermore, we confirmed the impact time of the participants from the high-speed camera (Figure 1A-⑥). The images from the scene camera of the eye-tracking device were recorded at a sampling frequency of 25 Hz, and the head and eye movements were recorded at a sampling frequency of 100 Hz.

### Data Analysis

To evaluate the gaze position, the gaze analysis software (Tobii Pro lab, Tobii Technology, Stockholm) was used to extract the gazing point. The gaze-ball angle was calculated using motion analysis software (Frame-DIASIV, DKH, Japan) based on images of gaze positions at the time when the ball bounced. These images were converted into two-dimensional data, and we digitized them to calculate the coordinates (pixel) of the ball and gaze position. After the digitizing process, the gaze position coordinates were subtracted from ball position coordinates to estimate the gaze-ball angle (Figure 1B). Finally, the pixel values of the coordinates were converted to angles based on the specifications of the eye-tracking device (horizontal angle: 82 deg/1920 px, vertical angle: 52 deg/1080 px). Through these processes, we obtained both the horizontal (Gaze-Ball Angle X) and vertical angles (Gaze-Ball Angle Y). Figure 2 shows a typical pattern of the gaze-ball angles in a single rally. These angles at the time the ball bounces (solid black line) were significantly different between the experts (blue) and semi-experts (red) players. Thus, we averaged the gaze-ball angle for three frames (pre-bounce, bounce, post-bounce). The gaze-ball angle was calculated as the mean absolute value for each participant.

The mean angular velocities of the pitch (downward and upward) and yaw (rightward and leftward) movements were calculated to evaluate the head movement during the rallies. At the same time, the mean angular velocities of the vertical (downward and upward) and horizontal (rightward and leftward) eye movements were calculated to evaluate the eye movement during the rallies. These data were filtered with 3rd order low pass filter using Jupyter Lab 3.2.1.

Figure 3 shows typical traces of the head angular position and velocity. The broken lines indicate the time when the experimenter hits the ball, and the vertical solid lines indicate the time when the participant hits the ball. The mean head angular velocity was calculated as the mean of the absolute values of a single trial (between the broken lines in Figure 3). The downward and rightward velocity phases correspond to the ball tracking phase (between the broken and solid lines in Figure 3), and the upward and leftward velocity phases correspond to the post-hitting phase (between the solid and broken lines in Figure 3).

Figure 4 shows typical eye position and velocity traces. The definitions of the vertical lines are the same as in Figure 3. The mean eye velocity was calculated as the mean values of a single trial (between the broken lines in Figure 4).

### Statistical Analysis

We compared the gaze-ball angle, head and eye velocities between the different subject groups using mixed-design analysis of variance with repeated measures with factors of the tempo sounds (130 and 150 bpm) and skill levels (experts and semi-experts). When a significant interaction was found, a simple main effect test was conducted for each level of the factors. When a significant main effect was found, a multiple comparison test using the Bonferroni method was conducted.

To examine the contribution of head and eye movements to the gaze-ball angle, a multiple regression analysis using stepwise regression was conducted with the gaze-ball angle as the dependent variable and head angular velocity and eye velocity as the independent variables. When the horizontal gaze-ball angle was the dependent variable, the rightward head angular velocity and eye velocity were the independent variables, and when the vertical gaze-ball angle was the dependent variable, the downward head angular velocity and eye velocity were the independent variables.

Statistical significance was set at *p* < 0.05 for all tests. Bonferroni adjustments were used for multiple comparisons. All statistical tests were conducted by IBM SPSS software version 27 (SPSS Inc, USA). Unless noted otherwise, data are presented as mean ± SD.

### Consistency of Experimental Conditions

We analyzed the hitting accuracy of the experimenter and the impact time to confirm the consistency of the experimental conditions. For the hitting accuracy of the experimenter, we calculated the distance (cm) from the center point of the circular target to the bounce point of the ball in all strokes by using a motion analysis software (Frame-DIASIV, DKH, Japan). A high-speed camera (frame rate = 120 fps, EX-ZR200, CASIO, Japan, Figure 1A-⑥) was used for capturing the bounce point of the ball. A black board with 42 white circles (radius: 1.0 cm) marked at 25.4 cm intervals was used to improve the accuracy of coordinate detection for the calibration. To confirm the consistency of the impact timing, we calculated the time (ms) from the impact of one player to that of another player based on the images of the high-speed camera (frame rate = 120 fps, EX-ZR200, CASIO, Japan, Figure 1A-⑥).

These data were calculated as the mean ± SD for each subject group in each tempo condition (Table 1). To examine the effect of the hitting accuracy of the experimenter and impact time on the main results, the Pearson correlation coefficient was calculated to examine the correlation between the hitting accuracy (distance) and the gaze-ball angle, eye and head movements, and between the impact time and the gaze-ball angle, eye and head movements. As a result, there was no significant relationship between them (Tables 2, 3). Therefore, this indicates that there is no effect of the hitting accuracy of the experimenter and impact time on the main results.

**Table 1:** Results of distance (A,B) and impact time (C,D) in each group.

	**Distance (cm)**		**Standard deviations (cm)**	
**A**			
Tempo sound	130	150	130	150
Experts	7.4	8.1	6.4	6.5
Semi-experts	7.2	8.5	6.2	6.4
**B**			
	**Distance SD (cm)**		**Standard deviations SD (cm)**	
Tempo sound	130	150	130	150
Experts	0.9	0.7	0.9	0.8
Semi-experts	1.2	1.2	1.4	1.1
**C**			
	**Impact time (ms)**		**Standard deviations (ms)**	
Tempo sound	130	150	130	150
Experts	459.4	398.8	15.6	15.5
Semi-experts	458	399.8	15.8	14.3
**D**			
	**Impact time SD (ms)**		**Standard deviations SD (ms)**	
Tempo sound	130	150	130	150
Experts	2.1	1.2	2.4	3.9
Semi-experts	2.7	4.3	2.3	4.1

**Table 2:** Pearson correlation between distance and the gaze-ball angle, eye velocity, and head velocity in each tempo.

**A**				**B**			
		**Distance (130 bpm)**				**SD (130 bpm)**	
		***r***	***p***			***r***	***p***
Gaze-ball angle	Horizontal	0.00	0.98	Gaze-ball angle	Horizontal	0.07	0.78
	Vertical	−0.16	0.51		Vertical	0.14	0.55
Head vel	Upward	0.37	0.11	Head vel	Upward	0.22	0.35
	Downward	0.43	0.07		Downward	0.07	0.78
	Rightward	0.21	0.38		Rightward	−0.11	0.64
	Leftward	0.12	0.60		Leftward	0.07	0.77
Eye vel	Upward	0.22	0.33	Eye vel	Upward	−0.10	0.68
	Downward	0.34	0.13		Downward	−0.24	0.30
	Rightward	0.13	0.56		Rightward	−0.15	0.52
	Leftward	0.13	0.57		Leftward	0.09	0.70
**C**				**D**			
		**Distance (150 bpm)**				**SD (150 bpm)**	
		***r***	***p***			***r***	***p***
Gaze-ball angle	Horizontal	−0.31	0.18	Gaze-ball angle	Horizontal	−0.19	0.43
	Vertical	−0.32	0.17		Vertical	−0.11	0.65
Head vel	Upward	0.07	0.76	Head vel	Upward	−0.03	0.89
	Downward	0.13	0.59		Downward	0.04	0.86
	Rightward	0.41	0.07		Rightward	0.27	0.26
	Leftward	0.39	0.09		Leftward	0.31	0.18
Eye vel	Upward	0.33	0.16	Eye vel	Upward	−0.01	0.98
	Downward	0.22	0.34		Downward	0.01	0.98
	Rightward	0.11	0.65		Rightward	−0.07	0.77
	Leftward	−0.25	0.29		Leftward	−0.35	0.12

**Table 3:** Pearson correlation between impact time and the gaze-ball angle, eye velocity, and head velocity in each tempo.

**A**				**B**			
		**Impact time (130 bpm)**				**SD (130 bpm)**	
		***r***	***p***			***r***	***p***
Gaze-ball angle	Horizontal	−0.26	0.27	Gaze-ball angle	Horizontal	−0.01	0.98
	Vertical	−0.26	0.26		Vertical	0.04	0.87
Head vel	Upward	0.40	0.08	Head vel	Upward	−0.16	0.51
	Downward	0.43	0.06		Downward	−0.11	0.63
	Rightward	0.33	0.16		Rightward	−0.18	0.45
	Leftward	0.35	0.17		Leftward	−0.31	0.18
Eye vel	Upward	0.23	0.32	Eye vel	Upward	0.07	0.77
	Downward	0.34	0.14		Downward	0.09	0.70
	Rightward	0.07	0.77		Rightward	0.14	0.56
	Leftward	0.36	0.17		Leftward	−0.14	0.54
**C**				**D**			
		**Impact time (150 bpm)**				**SD (150 bpm)**	
		***r***	***p***			***r***	***p***
Gaze-ball angle	Horizontal	−0.13	0.58	Gaze-ball angle	Horizontal	0.10	0.68
	Vertical	−0.20	0.40		Vertical	0.06	0.80
Head vel	Upward	0.00	1.00	Head vel	Upward	0.19	0.43
	Downward	0.02	0.95		Downward	0.20	0.40
	Rightward	0.17	0.47		Rightward	0.01	0.98
	Leftward	0.28	0.23		Leftward	0.14	0.54
Eye vel	Upward	0.44	0.05	Eye vel	Upward	0.37	0.11
	Downward	0.29	0.22		Downward	0.33	0.16
	Rightward	0.02	0.92		Rightward	0.35	0.13
	Leftward	−0.13	0.58		Leftward	0.15	0.53

In addition, we analyzed Pearson correlations between the height of participants and the gaze-ball angle, eye and head movements. As a result, there was no significant relationship between them (Table 4).

**Table 4:** Pearson correlation between the hight of participants and the gaze-ball angle, eye velocity, and head velocity in each tempo.

**A**			
**130 bpm**		**Hight**	
		***r***	***p***
Gaze-ball angle	Horizontal	0.24	0.43
	Vertical	0.25	0.41
Head vel	Upward	0.28	0.36
	Downward	−0.19	0.54
	Rightward	−0.27	0.37
	Leftward	−0.38	0.21
Eye vel	Upward	−0.07	0.81
	Downward	−0.29	0.34
	Rightward	−0.13	0.67
	Leftward	−0.25	0.41
**B**			
**150 bpm**		**Hight**	
		***r***	***p***
Gaze-ball angle	Horizontal	−0.01	0.99
	Vertical	0.14	0.65
Head vel	Upward	−0.45	0.15
	Downward	−0.24	0.43
	Rightward	−0.38	0.20
	Leftward	−0.51	0.08
Eye vel	Upward	−0.02	0.94
	Downward	−0.24	0.43
	Rightward	−0.19	0.54
	Leftward	−0.24	0.42

## Results

### Gaze-Ball Angle

The analysis of variance for the gaze-ball angle X showed that there were significant main effects for the subject group [*F*_(1,11)_ = 16.5, *p* < 0.01, *η*^2^ = 0.60, power = 0.96] and tempo condition [*F*_(1,11)_ = 88.7, *p* < 0.01, *η*^2^ = 0.89, power = 1.0]. Multiple comparison tests showed that the gaze-ball angle X at 130 bpm was significantly smaller than that at 150 bpm (*p* < 0.05). There was no significant interaction in any of the combinations (Figure 5A).

The analysis of variance for the gaze-ball angle Y showed that there were significant main effects for the subject group [*F*_(1,11)_ = 12.2, *p* < 0.01, *η*^2^ = 0.53, power = 0.89] and tempo condition [*F*_(1,11)_ = 38.1, *p* < 0.01, *η*^2^ = 0.78, power = 1.0]. Multiple comparison tests showed that the gaze-ball angle Y at 130 bpm was significantly smaller than that at 150 bpm (*p* < 0.05). There was no significant interaction in any of the combinations (Figure 5B).

### Eye and Head Movements

The analysis of variance for head movements did not show significant main effects for the subject group [rightward: *F*_(1,11)_ = 3.5, *p* = 0.09, *η*^2^ = 0.24, power = 0.40, leftward: *F*_(1,11)_ = 0.02, *p* = 0.89, *η*^2^ = 0.002, power = 0.05, downward: *F*_(1,11)_ = 4.2, *p* = 0.07, *η*^2^ = 0.27, power = 0.46, upward: *F*_(1,11)_ = 3.8, *p* = 0.12, *η*^2^ = 0.35, power = 0.61] and tempo condition [rightward: *F*_(1,11)_ = 3.4, *p* = 0.09, *η*^2^ = 0.24, power = 0.39, leftward: *F*_(1,11)_ = 3.0, *p* = 0.11, *η*^2^ = 0.22, power = 0.36, downward: *F*_(1,11)_ = 0.13, *p* = 0.73, *η*^2^ = 0.01, power = 0.06, upward: *F*_(1,11)_ = 1.1, *p* = 0.31, *η*^2^ = 0.09, power = 0.17]. There was no significant interaction in any of the combinations (Figure 6).

The analysis of variance for eye movements showed significant main effects for the subject group [rightward: *F*_(1,11)_ = 41.3, *p* < 0.01, *η*^2^ = 0.97, power = 1.0, leftward: *F*_(1,11)_ = 21.5, *p* < 0.01, *η*^2^ = 0.66, power = 0.98, downward: *F*_(1,11)_ = 23.0, *p* < 0.01, *η*^2^ = 0.68, power = 0.99, upward: *F*_(1,11)_ = 13.6, *p* < 0.01, *η*^2^ = 0.55, power = 0.92] and tempo condition [downward: *F*_(1,11)_ = 8.4, *p* < 0.05, *η*^2^ = 0.43, power = 0.75, upward: *F*_(1,11)_ = 8.8, *p* < 0.05, *η*^2^ = 0.45, power = 0.77]. There was no significant interaction in any of the combinations (Figure 7).

The multiple regression analysis described significant regression equations (Table 5A). There were significant negative correlations between the gaze-ball angle and head velocity only in the right direction (Figures 8A–D), and between the gaze-ball angle and eye velocity (Figures 9A–D) in each tempo condition. The valuables of head velocity in all conditions were excluded from equations by stepwise regression. The standardized coefficients (β) in all conditions are shown in Table 5B. The standardized coefficients of the eye velocity were significantly higher than that of head velocity. The VIFs were all <10.0 and there was no problem with multicollinearity. These results indicate that the effect of eye velocity on the gaze-ball angle is significantly higher than that of head velocity.

**Table 5:** Results of multiple regression analysis. (A) Significance of the regression equations, (B) Standardized coefficients.

**A**						
	**Independent valueable**	**Dependent valueable**	**Adjusted *R*^2^**	**DOF**	***F***	***p***
130 bpm	Gaze-ball angle X	Right head vel	0.62	1, 11	20.4	<0.01
		Right eye vel				
	Gaze-ball angle Y	Down head vel	0.56	1, 11	16.3	<0.01
		Down eye vel				
150 bpm	Gaze-ball angle X	Right head vel	0.6	1, 11	19.1	<0.01
		Right eye vel				
	Gaze-ball angle Y	Down head vel	0.45	1, 11	10.6	<0.01
		Down Eye Vel				
**B**						
	**Independent valueable**	**Dependent valueable**	***β***	***t***	***p***	**VIF**
130 bpm	Gaze-ball angle X	Right head vel	−0.28	−1.1	0.29	2.0
		Right eye vel	−0.81	−4.5	<0.01	1.0
	Gaze-ball angle Y	Down head vel	0.14	0.57	0.58	1.6
		Down eye vel	−0.78	−4.0	<0.01	1.0
150 bpm	Gaze-ball angle X	Right head vel	−0.31	−1.3	0.24	2.0
		Right eye vel	−0.80	−4.4	<0.01	1.0
	Gaze-ball angle Y	Down head vel	0.11	0.37	0.72	1.6
		Down eye vel	−0.70	−3.3	<0.01	1.0

## Discussion

In this study, we examined the properties of visual strategies in table tennis players of different skill levels while they conducted rallies. We found significant differences in the gaze-ball angle, and eye movements between the expert and semi-expert groups. This finding indicates that the visual strategies during table tennis rallies are different between the expert and semi-expert players. Furthermore, the multiple regression analysis showed that the effect of eye velocity on the gaze-ball angle was significantly higher than that of head velocity. Our results suggest that eye movements are more tightly associated with gaze position than head movements during table tennis rallies.

### Gaze-Ball Angle

The gaze-ball angle of the expert players in each direction was larger than that of the semi-expert players. This finding indicates that the visual strategy of expert players was different from that of semi-expert players during fast rallies. Concretely, this result reflects that the expert players tend to keep watching the ball shorter than the semi-expert players during rallies. This result supports the previous results reported by Ishigaki (2007). Moreover, the higher the tempo, the larger the gaze-ball angle. Therefore, our result suggests that the higher the tempo, the earlier table tennis players take their gaze position away from the ball in ball-tracking. The advantage of the large gaze-ball angle is that the player can acquire not only the ball, but also surround visual information such as the opposite player. Understanding the stroke motion of the opposite player during rallies is important to determine the stroke timing and predict ball speed.

There are several previous studies on visual strategy in terms of eye and head movements during interceptive sports, such as table tennis (Ripoll and Fleurance, 1988; Rodrigues et al., 2002), cricket (Croft et al., 2010; Mann et al., 2013), and baseball (Higuchi et al., 2018; Kishita et al., 2020a,b). However, the results of this study regarding the gaze-ball angle of the expert table tennis players in the ball-tracking phase have never been demonstrated. Higuchi et al. (2016) have suggested that the hitting performance does not change even though there is no visual information of the ball flight for the latest 150 ms before the time of ball-bat contact. Although the expert players in this study did not watch the ball clearly for 100–200 ms before the ball hit the racket, they could be able to predict the ball trajectory for successful hitting. Nakamoto et al. (2015) suggests that it is possible that expert players control motor timing based on the future target trajectory constructed by the brain without acquiring online visual information. Since the expert players in this study had a mean of 13.8 years of table tennis experience, their brains might have predictive visual perception mechanisms obtained through training.

### Eye and Head Movements

There was no difference in head velocity between the subject groups. This finding suggests that head movements are not associated with competition levels. Our results are consistent with a previous study showing individual differences in head movement when pursuing the ball in baseball batting (Higuchi et al., 2018). In addition to the result obtained by Higuchi et al. (2018), head movements may not have a characteristic observed in the skilled player's performance. Furthermore, the contribution of head velocity was significantly lower than that of eye velocity as the result of multiple regression analyses. This result suggests that head velocity is not associated with the gaze position based on visual strategy during rallies. Kishita et al. (2020b) have reported that eye and head movements for baseball batting are determined temporally in relation to body movements. Therefore, in table tennis situations, it is also possible that head movements are related to body movements independent of visual strategy.

In contrast to the head velocity, there were significant differences in the eye velocity between the subject groups. Concretely, the eye velocity of the expert players was significantly smaller than that of the semi-expert players. These results reflect that the gaze-ball angle of expert players is significantly larger than that of semi-expert players. The advantage of the small eye velocity is also to acquire visual information about surroundings other than the ball, as well as the large gaze-ball angle. This result is also consistent with a previous study by Higuchi et al. (2018) suggesting that “excessive changes in the eyes” position may increase the batter's chance of mis-hitting the ball. They also suggest that the eye movements of skilled baseball players are more stable during the ball tracking phase. Therefore, the small velocity of eye movement could contribute to the successful performance in regular tempo rallies. In addition, the result of multiple regression analysis showed that the effect of eye velocity on the gaze-ball angle was significantly higher than that of head velocity. Thus, it is most likely that eye velocity is associated with the gaze position based on visual strategies during rallies. It is well-known that in gaze control, eye and head movements interact with each other. However, our study demonstrated that gaze angle during table tennis rallies was associated with eye movements rather than head movement. This result is consistent with Kishita et al. (2020b) showing that head movement during rallies is affected by stroke movements. Therefore, eye movements may contribute to gaze position more than head movements, while head movements could be more associated with stroke movements during table tennis rallies. We have clarified the description in the discussion to address this point.

Furthermore, there were significant differences in the vertical eye velocity between the different tempo conditions. This result indicates that vertical eye movements are affected by the tempo conditions. Although there are few previous studies that assessed eye and head movements in a vertical direction during the interceptive performance (Land and McLeod, 2000; Mann et al., 2019; Fogt and Persson, 2020), the present study is the first study to quantitatively clarify significant differences in vertical eye movements between two different skill levels. The tendency of the vertical eye movement is similar to that of horizontal eye movement, the head moves obliquely during a table tennis rally, which often induces horizontal and vertical eye and head motion, simultaneously.

## Conclusion

Visual strategies of the two different table tennis players groups during fast rallies were examined. The results showed that the gaze-ball angle of expert players was significantly larger than that of semi-expert players. We also found that the eye velocity of expert players was significantly smaller than that of semi-expert players. These results indicate significant differences in gaze position and eye movements between the competition levels. Furthermore, the effect of eye velocity on the gaze-ball angle was significantly higher than that of head velocity, regardless of competition level. Our results mentioned above suggest that the visual strategies in table tennis rallies are associated with eye movements rather than head movements.

## Data Availability Statement

The raw data supporting the conclusions of this article will be made available by the authors, without undue reservation.

## Ethics Statement

The studies involving human participants were reviewed and approved by Research Ethics Committee at the Faculty of Health and Sport Sciences, University of Tsukuba. The patients/participants provided their written informed consent to participate in this study.

## Author Contributions

RS, SA, YN, TK, and SO conceived and designed the experiments. RS, TK, and SO interpreted the data. RS performed the experiment, conducted the data analysis, and drafted the manuscript. SA, YN, TK, and SO edited, revised the manuscript, and approved the final version. All authors contributed to the article and approved the submitted version.

## Funding

This research was supported in part by JSPS KAKENHI Grant Numbers 19K11460 and 22H03492.

## Conflict of Interest

The authors declare that the research was conducted in the absence of any commercial or financial relationships that could be construed as a potential conflict of interest.

## Publisher's Note

All claims expressed in this article are solely those of the authors and do not necessarily represent those of their affiliated organizations, or those of the publisher, the editors and the reviewers. Any product that may be evaluated in this article, or claim that may be made by its manufacturer, is not guaranteed or endorsed by the publisher.

## References

[B1] Akpinar S. Devrilmez E. Kirazci S. (2012). Coincidence-anticipation timing requirements are different in racket sports. Percept. Mot. Skills 115, 581–593. 10.2466/30.25.27.PMS.115.5.581-593 23265020

[B2] Croft J. L. Button C. Dicks M. (2010). Visual strategies of sub-elite cricket batsman in response to different ball velocities. Hum. Mov. Sci. 29, 751–763. 10.1016/j.humov.2009.10.004 20031242

[B3] Faber I. R. Oosterveld F. G. J Sanden N. (2014). Does an eye-hand coordination test have added value as part of talent identification in table tennis? A validity and reproducibility study. PLoS ONE. 9:e85657. 10.1371/journal.pone.0085657 24465638 PMC3895002

[B4] Fogt N. Persson T. W. (2020). Vertical head and eye movements in baseball batting. Optom. Vis. Perform. 8, 129–134. 33283000 PMC7717490

[B5] Higuchi T. Nagami T. Nakata H. Kanosue K. (2018). Head-eye movement of collegiate baseball batters during fastball hitting. PLoS ONE 13:e0200443. 10.1371/journal.pone.0200443 30016367 PMC6049917

[B6] Higuchi T. Nagami T. Nakata H. Watanabe M. Isaka T. Kanosue K. (2016). Contribution of visual information about ball trajectory to baseball hitting accuracy. PLoS ONE 11:e0148498. 10.1371/journal.pone.0148498 26848742 PMC4743964

[B7] Ishigaki H. (2007). Difference in line of sight during table tennis railies due to performance level. Bulletin of Aichi Institute of Technology. B 167–170.

[B8] Kidokoro S. Inaba Y. Yoshida K. Yamada K. Ozaki H. (2019). The influence of shoulder and torso joint mobility on three different topspin forehands in a situation accompanied by body movement in Japanese elite table tennis players. Japan J. Phys. Educ. Health. Sport Sci. 64, 169–185. 10.5432/jjpehss.18029

[B9] Kishita Y. Ueda H. Kashino M. (2020a). Eye and head movements of elite baseball players in real batting. Front. Sports Act Living. 2:3. 10.3389/fspor.2020.00003 33344998 PMC7739578

[B10] Kishita Y. Ueda H. Kashino M. (2020b). Temporally coupled coordination of eye and body movements in baseball batting for a wide range of ball speeds. Front. Sports Act Living. 2:64. 10.3389/fspor.2020.00064 33345055 PMC7739824

[B11] LaFont D. (2007). Watch the Ball? ITF Coaching and Sport Science Review 43:11.

[B12] Land M. F. McLeod P. (2000). From eye movements to actions: how batsman hit the ball. Nat. Neurosci. 3, 1340–1345. 10.1038/81887 11100157

[B13] Mann D. L. Nakamoto H. Logt N. Sikkink L. Brenner E. (2019). Predictive eye movements when hitting a bouncing ball. J. Vis. 19:28. 10.1167/19.14.28 31891654

[B14] Mann D. L. Spratford W. Abernethy. (2013). The head tracks and gaze predicts: how the world's best batters hit a ball. PLoS ONE 8:e58289. 10.1371/journal.pone.0058289 23516460 PMC3596397

[B15] Mansec Y. L. Dorel S. Nordez A. Jubeau M. (2016). Sensitivity and reliability of a specific test of stroke performance in table tennis. Int. J. Physiol. Perform. 11, 678–684. 10.1123/ijspp.2015-0444 26640961

[B16] Nakamoto H. Mori S. Ikudome S. Uenaka S. Imanaka K. (2015). Effects of sport expertise on representational momentum during timing control. Atten. Percept. Psychophys. 77, 961–971. 10.3758/s13414-014-0818-9 25537739

[B17] Nakata H. Miura A. Yoshie M. Kudo K. (2012). Differences in the head movement during baseball batting between skilled players and novices. J. Strength Cond. Res. 26, 2632–2640. 10.1519/JSC.0b013e3182429c38 22130405

[B18] Pallus A. P. Freedman E. G. (2016). Target position relative to the head is essential for predicting head movement during head-free gaze pursuit. Exp. Brain Res. 234, 2107–2121. 10.1007/s00221-016-4612-x 26979437 PMC5058444

[B19] Ripoll H. Fleurance P. (1988). What does keeping one' s eye on the ball mean? Ergonomics 31, 1647–1654. 10.1080/00140138808966814 3229411

[B20] Rodrigues S. T Vickers J. N Williams A. M. (2002). Head eye and arm coordination in table tennis. J. Sports Sci. 20, 187–200. 10.1080/026404102317284754 11999475

[B21] Roy J. E. Cullen K. E. (2003). Brain stem pursuit pathways: dissociating visual, vestibular, and proprioceptive inputs during combined eye-head gaze tracking. J. Neurophysiol. 90, 271–290. 10.1152/jn.01074.2002 12843311

[B22] Sheppard A. Li F. (2007). Expertise and the control of interception in table tennis. Eur. J. Sport Sci. 7, 213–222. 10.1080/17461390701718505

